# The different overall survival between single-agent EGFR-TKI treatment and with bevacizumab in non-small cell lung cancer patients with brain metastasis

**DOI:** 10.1038/s41598-022-08449-w

**Published:** 2022-03-15

**Authors:** Tzu-Hsuan Chiu, Pi-Hung Tung, Chi-Hsien Huang, Jia-Shiuan Ju, Allen Chung-Cheng Huang, Chin-Chou Wang, Ho-Wen Ko, Ping-Chih Hsu, Yueh-Fu Fang, Yi-Ke Guo, Chih-Hsi Scott Kuo, Cheng-Ta Yang

**Affiliations:** 1 Division of Thoracic Oncology, Department of Thoracic Medicine, College of Medicine, Chang Gung Memorial Hospital, Chang Gung University, Taipei, Taiwan; 2 Thoracic Oncology Unit, Chang Gung Memorial Hospital Cancer Center, Taipei, Taiwan; 3 Division of Pulmonary and Critical Care Medicine, Kaohsiung Chang Gung Memorial Hospital, Kaohsiung, Taiwan; 4 Department of Computing, Data Science Institute, Imperial College London, London, UK

**Keywords:** Cancer, Lung cancer, Non-small-cell lung cancer

## Abstract

Comparison of epidermal growth factor receptor tyrosine kinase inhibitor (EGFR-TKI) monotherapy or with bevacizumab in real-world non-small cell lung cancer (NSCLC) patients was lacking. 310 patients of advanced NSCLC with common *EGFR* mutation receiving first-generation EGFR-TKI monotherapy or with bevacizumab were included and propensity-score matched. Progression-free survival (PFS), overall survival (OS) and secondary T790M mutation were analysed. Patients receiving EGFR-TKI and bevacizumab were significantly younger, had better performance status and with high incidence of brain metastasis (55.8%). In the propensity-score matched cohort, PFS (13.5 vs. 13.7 months; log-rank p = 0.700) was similar between the two groups. The OS (61.3 vs. 34.2 months; log-rank p = 0.010) and risk reduction of death (HR 0.42 [95% CI 0.20–0.85]; p = 0.017) were significantly improved in EGFR-TKI plus bevacizumab group. Analysis of treatment by brain metastasis status demonstrated EGFR-TKI plus bevacizumab in patients with brain metastasis was associated with significant OS benefit compared to other groups (log-rank p = 0.030) and these patients had lower early-CNS and early-systemic progressions. The secondary T790M did not significantly differ between EGFR-TKI plus bevacizumab and EGFR-TKI monotherapy groups (66.7% vs. 75.0%, p = 0.460). Forty-one (31.1%) and 31 (23.5%) patients received subsequent osimertinib and chemotherapy, respectively. The post-progression OS of osimertinib and chemotherapy were 22.1 and 44.9 months in EGFR-TKI plus bevacizumab group and were 10.0 and 14.1 months in EGFR-TKI monotherpay group, respectively. First-generation EGFR-TKI with bevacizumab improved treatment efficacy in real-world patients of NSCLC with *EGFR* mutation. Patients with brain metastasis received additional OS benefit from this treatment.

## Introduction

Erlotinib and gefitinib are first-generation EGFR-TKIs with non-covalent and reversible binding activity to receptor tyrosine kinase and are both approved treatments for patients of advanced NSCLC harboring sensitizing *EGFR* mutation^1,2^. Although patients treated by first-generation EGFR-TKI demonstrated an improved efficacy compared to platinum-based chemotherapy, this treatment usually exhibited variable objective response rates at approximately 50–75% and a median PFS between 9 to14 months^3–6^.

A number of combination strategies were attempted in the hope to improve the treatment efficacy of single-agent EGFR-TKI. This includes anti-angiogenesis agents that specifically target vascular endothelial growth factor (VEGF) or VEGF receptor 2 (VEGFR2)^7^. The long-established role of angiogenesis in the progression of lung cancer has supported the rationale to combine anti-angiogenesis agents with active anti-cancer treatments^8^. Two previous randomized studies, the JO25567 and NEJ026 trials, which mainly involved Japanese patients, have demonstrated PFS benefit of erlotinib plus bevacizumab compared to erlotinib monotherapy in the front-line treatment of advanced NSCLC with sensitizing *EGFR* mutation^9,10^. However, the OS benefit from bevacizumab add-on was not observed in these studies which may be partly related to the cross-over design of the trials.

The superior efficacy of erlotinib and anti-angiogenesis combination over single-agent erlotinib did not seem to be consistent across different clinical trials. Stinchcombe et al. conducted a phase II randomized study comparing erlotinib monotherapy and with bevacizumab in a Caucasian cohort of *EGFR*-mutant NSCLC patients^11^. In this study, PFS and OS were similar in the two groups. Ichihara et al. reported another phase II single-arm study on gefitinib and bevacizumab combination. The study did not meet the primary endpoint of 1-year PFS though a seemingly longer PFS than historical data of gefitinib monotherapy was observed^12^.

Previously, administration of bevacizumab has demonstrated clinical benefit for both primary brain tumor and brain metastasis from advanced NSCLC, likely as a result of suppressing tumor angiogenesis and reducing intracranial vasogenic edema^13,14^. Lately, significantly improved OS of bevacizumab treatment in advanced NSCLC patients with brain metastasis compared to those without was also observed in a real-world US cancer registry and claim database^15^. Thus, the survival benefit of bevacizumab treatment in *EGFR*-mutant NSCLC patients with brain metastasis warrants further investigation.

Currently, the third-generation EGFR-TKI osimertinib has arisen as a preferred first-line treatment option for advanced *EGFR*-mutant NSCLC^16,17^. Recently, combination of osimertinib and bevacizumab has been investigated in both second-line and first-line setting. Akamatsu et al. demonstrated that efficacy of osimertinib plus bevacizumab is statistically equivalent to, yet numerically lower than, osimertinib monotherapy in patients of acquired *EGFR* T790M-positive NSCLC^18^. In contrary, a phase I/II single arm study reported by Yu et al. showcased a positive result in which osimertinib plus bevacizumab in treatment-naïve *EGFR*-mutant NSCLC patients met the pre-specified effectiveness end point^19^. On the other side, treatment of osimertinib plus ramucirumab has also been investigated in two phase I studies whereas the efficacy data was premature^20,21^. Overall, whether combination of third-generation EGFR-TKI and anti-angiogenesis agents will be a new standard of treatment remains unsettled.

In this study, we analysed a real-world, brain metastasis-enriched cohort of NSCLC patients with *EGFR*-sensitizing mutation who received first-line erlotinib/gefitinib monotherapy or with bevacizumab. The treatment efficacy and the development of secondary T790M were compared between the two groups.

## Methods

### Patients and treatment

Patients who received a first-generation EGFR-TKI (gefitinib or erlotinib) monotherapy or with bevacizumab as the first-line treatment of advanced NSCLC with common *EGFR* mutation (exon 19 deletion or exon 21 L858R) were retrospectively included from January 2014 to December 2019. The dose of EGFR-TKI administered were gefitinib 250 mg/day and erlotinib 150 mg/day, respectively; and bevacizumab was administered at a dose of 7.5 mg/kg every 3 weeks. The lower dose strength was chosen because bevacizumab is not reimbursed by National Health Insurance of Taiwan for NSCLC treatment and this dosage has been demonstrated to achieve an equivalent efficacy and numerically lower adverse events compared to the 15 mg/kg dose strength^22,23^ Patients were excluded if the first dose of bevacizumab was given 3 weeks behind the first dose of EGFR-TKI and those who received an EGFR-TKI monotherapy less than 14 days were also excluded. The progression-free survival (PFS) was defined as the interval between the date of starting EGFR-TKI treatment and the date of radiologically or clinically determined progression or death. The treatment response, including complete response (CR), partial response (PR), stable disease, and progressive disease, was evaluated according to the Response Evaluation Criteria in Solid Tumors (version 1.1). The study used data from the Chang Gung Research Database and the study protocol and the waiver of informed consent form were approved by the Ethics Committee of Chang Gung Memorial Hospital.

### Statistical analysis

The Mann–Whitney test was used to determine the statistical significance of continuous variables between the two groups and Fisher exact test was used for evaluating the categorical variables. The Kaplan–Meier survival curves were generated using the R package *survival*, and the hazard ratio (HR) was analysed using the Cox regression model. The propensity-score-matched analysis was used to balance the clinical characteristics between the treatment groups as previously described, in which the distance measure was defined by generalized linear model, the matching method was nearest neighbor matching and the caliper was 0.1 in standard deviation unit^6^. Briefly, the EGFR-TKI plus bevacizumab and EGFR-TKI monotherapy groups served as the dependent variables and the covariates used included age, ECOG PS, *EGFR* mutation subtypes, brain metastasis and type of EGFR-TKI administered. Paired patients treated with EGFR-TKI plus bevacizumab or EGFR-TKI alone with equivalent propensity scores were selected in a 1:2 manner using the R package *MatchIt*. The disease progression patterns (CNS progression alone without death versus systemic progression without death) were treated as competing risk events of which the cumulative incidence functions were calculated. The modified Cox regression model for the subdistribution hazard of the cumulative incidence function was applied to calculate the disease progression hazard from a given pattern in the presence of competing events by using the R package *cmprsk*. All reported *p*-values are two sided; *p* < 0.05 was considered statistically significant. Data were also analysed using SPSS (version 10.1; SPSS, Chicago, IL, USA).

### Ethics statement

The study was performed in accordance with the ethical standards of the 1964 Declaration of Helsinki. The Ethics Committee of Chang Gung Memorial Hospital approved the study (No. 201801967B0) and granted permission for access to the Chang Gung Research Database and approved the waiver of the informed consent form.

## Results

### Baseline patient characteristics

Overall, 310 patients were included in present study in which 267 (86.1%) patients received the treatment of single-agent EGFR-TKI and 43 (13.9%) patients received the treatment of EGFR-TKI plus bevacizumab. Compared to patients who received EGFR-TKI monotherapy, those who received bevacizumab combination in real-world practice were significantly younger (60.4 ± 9.8 vs. 71.2 ± 12.1; p < 0.001), more likely to have erlotinib as treatment partner (90.7% vs. 55.1%; p < 0.001), have a better performance status (ECOG PS 0–1 88.4% vs. 72.7%; p = 0.036) and more likely to present a brain metastasis (55.8% vs. 42.3%; p = 0.140, Table 1). The other patient characteristics including smoking status, sex, *EGFR* mutation subtype and liver metastasis were similar in the two groups.

**Table 1:** Overall patient characteristics.

	Total (%) N = 310	TKI plus bevacizumab (%) N = 43	TKI alone (%) N = 267	p value
Age (mean ± SD)		60.4 ± 9.8	71.2 ± 12.1	< 0.001
Age ≥ 65	208 (67.1)	15 (34.9)	193 (72.3)	< 0.001
ECOGPS 0–1	232 (74.8)	38 (88.4)	194 (72.7)	0.036
**Gender**				
Male	105 (33.9)	14 (32.6)	237 (34.1)	1.000
Current/ex-smoker	73 (23.5)	11 (25.6)	62 (23.2)	0.703
**Histology**				
Adenocarcinoma	307 (99.0)	43 (100.0)	264 (98.9)	1.000
Others	3 (1.0)	0	3 (1.1)	
***EGFR* mutation**				
L858R	185 (59.7)	23 (53.5)	162 (60.7)	0.405
19deletion	125 (40.3)	20 (46.5)	105 (39.3)	
**Disease stage**				
III	18 (5.6)	1 (2.3)	17 (6.4)	0.485
IV	292 (94.4)	42 (97.7)	250 (93.6)	
**Site of metastasis**				
Brain	137 (44.2)	24 (55.8)	113 (42.3)	0.140
Liver	40 (12.6)	7 (16.3)	33 (12.4)	0.469
**EGFR TKI administered**				
Gefitinib	124 (40.0)	4 (9.3)	120 (44.9)	< 0.001
Erlotinib	186 (60.0)	39 (90.7)	147 (55.1)	

### Cox regression analyses of overall survival in all patients

Cox regression survival analyses were performed to determine the independent factors that associated with OS in all study subjects. In univariate analyses, ECOG PS 0–1 (HR 0.44 [95% CI 0.31–0.62]; *p* < 0.001) and bevacizumab treatment (HR 0.41 [95% CI 0.21–0.78]; *p* = 0.006) were associated with a prolonged OS whereas *EGFR* L858R mutation (HR 1.49 [95% CI 1.06–2.09]; *p* = 0.022) and liver metastasis (HR 1.43 [95% CI 0.89–2.31]; *p* = 0.139, Table 2) were associated with a reduced OS. In multivariate analyses, ECOG PS 0–1 (HR 0.46 [95% CI 0.33–0.66]; *p* < 0.001) and bevacizumab treatment (HR 0.48 [95% CI 0.25–0.92]; *p* = 0.027) remained predictive of an improved OS. On the other side, *EGFR* L858R mutation (HR 1.42 [95% CI 1.01–2.02]; *p* = 0.047, Table 2) still served as a negative predictor of OS.

**Table 2:** Cox regression overall survival analysis.

Variable	Univariate analysis			Multivariate analysis		
	HR	95% CI	p value	HR	95% CI	p value
Age ≥ 65	1.22	0.86–1.73	0.259	–	–	–
ECOG 0, 1	0.44	0.31–0.62	< 0.001	0.46	0.33–0.66	< 0.001
Male	1.24	0.89–1.73	0.196	–	–	–
Current/ex-smoker	1.30	0.91–1.87	0.152	–	–	–
*EGFR* L858R	1.49	1.06–2.09	0.022	1.42	1.01–2.02	0.047
Bevacizumab treatment	0.41	0.21– 0.78	0.006	0.48	0.25–0.92	0.027
Brain metastasis	1.15	0.83–1.60	0.397			
Liver metastasis	1.43	0.89–2.31	0.139	1.20	0.73–1.96	0.464

### Treatment outcomes in the propensity-score matched patients

**Figure 1:**
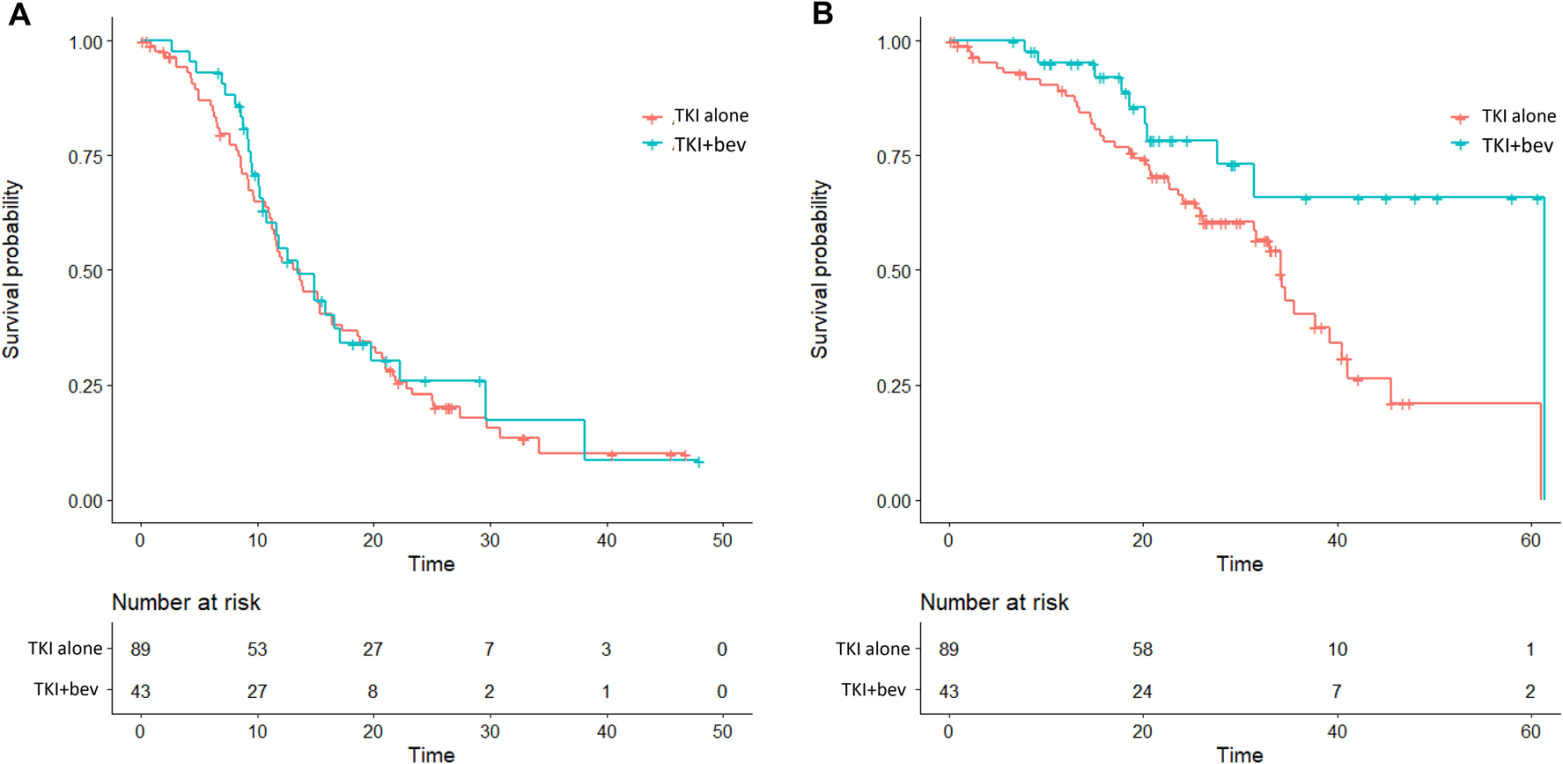
(**A**) PFS and (**B**) OS of EGFR-TKI plus bevacizumab and EGFR-TKI monotherapy groups. *bev* bevacizumab, *TKI* tyrosin kinase inhibitor.

**Figure 2:**
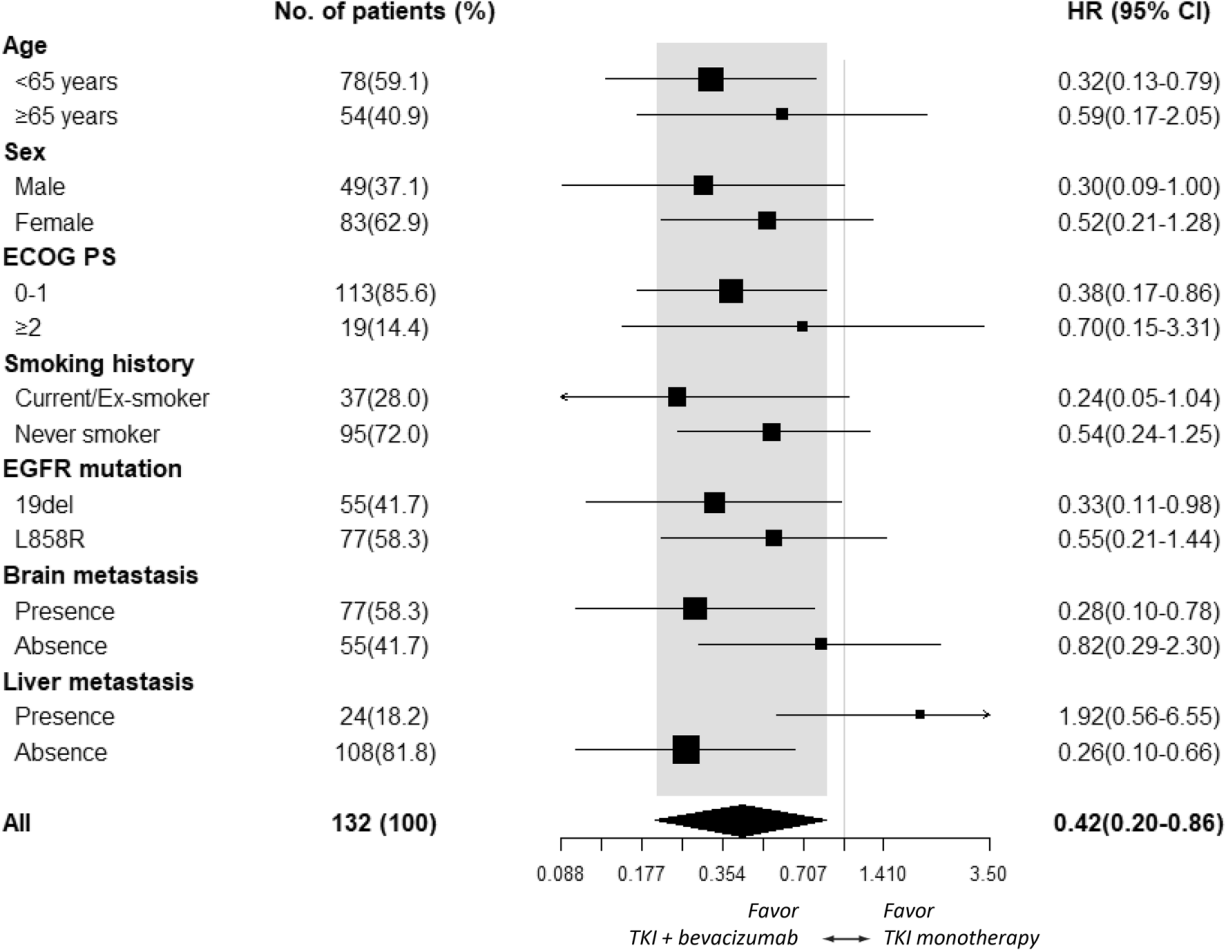
Subgroup analysis of OS of EGFR-TKI plus bevacizumab and EGFR-TKI monotherapy groups.

A propensity-score matching was subsequently conducted in a 1:2 manner between the EGFR-TKI plus bevacizumab and EGFR-TKI monotherapy groups. After matching, a cohort of 132 patients with balanced characteristic was attained which consisted of 43 and 89 patients in EGFR-TKI plus bevacizumab group and single-agent EGFR-TKI group, respectively (Table 3). The median follow-up duration, estimated by reverse Kaplan–Meier method, was 23.1 months and 32.9 months in the EGFR-TKI plus bevacizumab and EGFR-TKI monotherapy groups, respectively. The median cycle of bevacizumab treatment was 11 (7.0–17.5). At the end of follow up, 29 (67.4%) events of disease progression or death were noted in the EGFR-TKI plus bevacizumab group and 69 (77.5%) events were observed in the EGFR-TKI monotherapy group. The median PFS (13.5 vs. 13.7 months; log-rank test p = 0.700), risk reduction toward disease progression (HR 0.90 [95% CI 0.58–1.40]; p = 0.656) and the 24-month PFS rate (26.0% [95% CI 14.3–47.0%] vs. 22.9% [95% CI 15.3–34.2%], Fig. 1A) were similar between the two groups. However, the median OS (61.3 vs. 34.2 months; log-rank test p = 0.010), risk reduction of death (HR 0.42 [95% CI 0.20–0.85]; p = 0.017) and the 3-year OS rate (65.8% [95% CI 48.3–89.6%] vs. 40.5% [95% CI 28.8–56.8%], Fig. 1B) was significantly improved in the EGFR-TKI plus bevacizumab group compared the single-agent EGFR-TKI group. Subgroup OS analysis indicated additional benefit of bevacizumab in patients with brain metastasis (HR 0.28 [95% CI 0.10–0.78]; p = 0.015), patients who had ECOG PS 0–1 (HR 0.38 [95% CI 0.17–0.86]; p = 0.020), patients with *EGFR* 19 deletion mutation (HR 0.33 [95% CI 0.11–0.98]; p = 0.046) and patients who had no liver metastasis (HR 0.26 [95% CI 0.10–0.66]; p = 0.005, Fig. 2).

**Table 3:** Propensity-score matched cohort.

	Total (%) N = 132	TKI plus bevacizumab (%) N = 43	TKI alone (%) N = 89	p value
Age (mean ± SD)		60.4 ± 9.8	62.4 ± 10.6	0.284
Age ≥ 65	54 (40.9)	15 (34.9)	39 (43.8)	0.351
ECOG PS 0–1	113 (85.6)	38 (88.4)	75 (84.3)	0.606
**Gender**				
Male	49 (37.1)	14 (32.6)	35 (39.3)	0.565
Current/ex-smoker	37 (28.0)	11 (25.6)	26 (29.2)	0.836
**Histology**				
Adenocarcinoma	132 (100.0)	43 (100.0)	89 (100.0)	1.000
***EGFR* mutation**				
L858R	77 (58.3)	23 (53.5)	54 (60.7)	0.456
19deletion	55 (41.7)	20 (46.5)	35 (39.3)	
**Disease stage**				
III	1 (0.8)	1 (2.3)	0	0.326
IV	131 (99.2)	42 (97.7)	89 (100.0)	
**Site of metastasis**				
Brain	77 (58.3)	24 (55.8)	53 (59.6)	0.710
Liver	24 (18.2)	7 (16.3)	17 (19.1)	0.812
**EGFR TKI administered**				
Gefitinib	13 (9.8)	4 (9.3)	9 (10.1)	1.000
Erlotinib	119 (90.2)	39 (90.7)	80 (89.9)	
**Local treatment of brain metastasis**				
WBRT	38 (28.8)	10 (23.3)	28 (31.5)	0.325
SRS	1 (0.8)	1 (2.3)	0	
Surgical resection	9 (6.8)	4 (9.3)	5 (5.6)	

*WBRT* whole brain radiotherapy, *SRS* stereotactic radiosurgery.

### Patients of brain metastasis and pattern of disease progression

**Figure 3:**
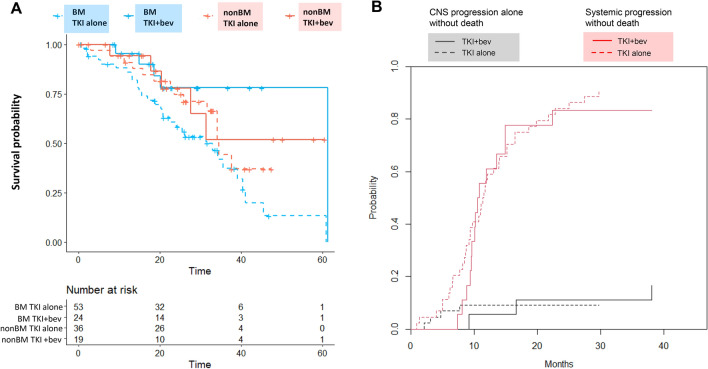
(**A**) OS of EGFR-TKI plus bevacizumab and EGFR-TKI monotherapy by the baseline status of brain metastasis (**B**) Cumulative incidence of systemic progression without death (red) and CNS progression alone without death (black) between EGFR-TKI plus bevacizumab and EGFR-TKI monotherapy groups in patients with baseline brain metastasis. *bev* bevacizumab, *BM* brain metastasis, *TKI* tyrosin kinase inhibitor.

The OS of EGFR-TKI plus bevacizumab and EGFR-TKI monotherapy by the status of baseline brain metastasis was analysed. Patients with brain metastasis who received EGFR-TKI plus bevacizumab and EGFR-TKI monotherapy demonstrated the median OS (61.3 vs. 33.0 months) and the 3-year OS rate (78.1% [95% CI 61.1–99.9%] vs. 37.2% [95% CI 23.6–58.6%]), respectively. In patients without brain metastasis, the median OS (not reach vs. 34.6 months) and the 3-year OS rate (51.9% [95% CI 27.5–98.1%] vs. 44.3% [95% CI 26.0–75.3%], Fig. 3A) were noted in EGFR-TKI plus bevacizumab and single-agent EGFR-TKI groups. A significant OS difference was observed among the four treatment groups (Fig. 3A; log-rank test p = 0.030). The pattern of disease progression in patients with baseline brain metastasis was analysed in terms of the cumulative incidence of systemic or CNS progression. The rates of CNS progression (cause-specific HR, 1.77; 95% CI 0.41–7.56; p = 0.858) and of systemic progression over time (cause-specific HR, 0.88; 95% CI 0.49–1.60; p = 0.692, Fig. 3B) did not differ significantly between EGFR-TKI plus bevacizumab and single-agent EGFR-TKI groups whereas treatment of EGFR-TKI plus bevacizumab was associated with lower emergence of early-CNS and early-systemic progressions which were both delayed to occur beyond 8 to 9 months of treatment (Fig. 3B).

### Acquired T790M mutation and efficacy of subsequent post-progression treatment

**Figure 4:**
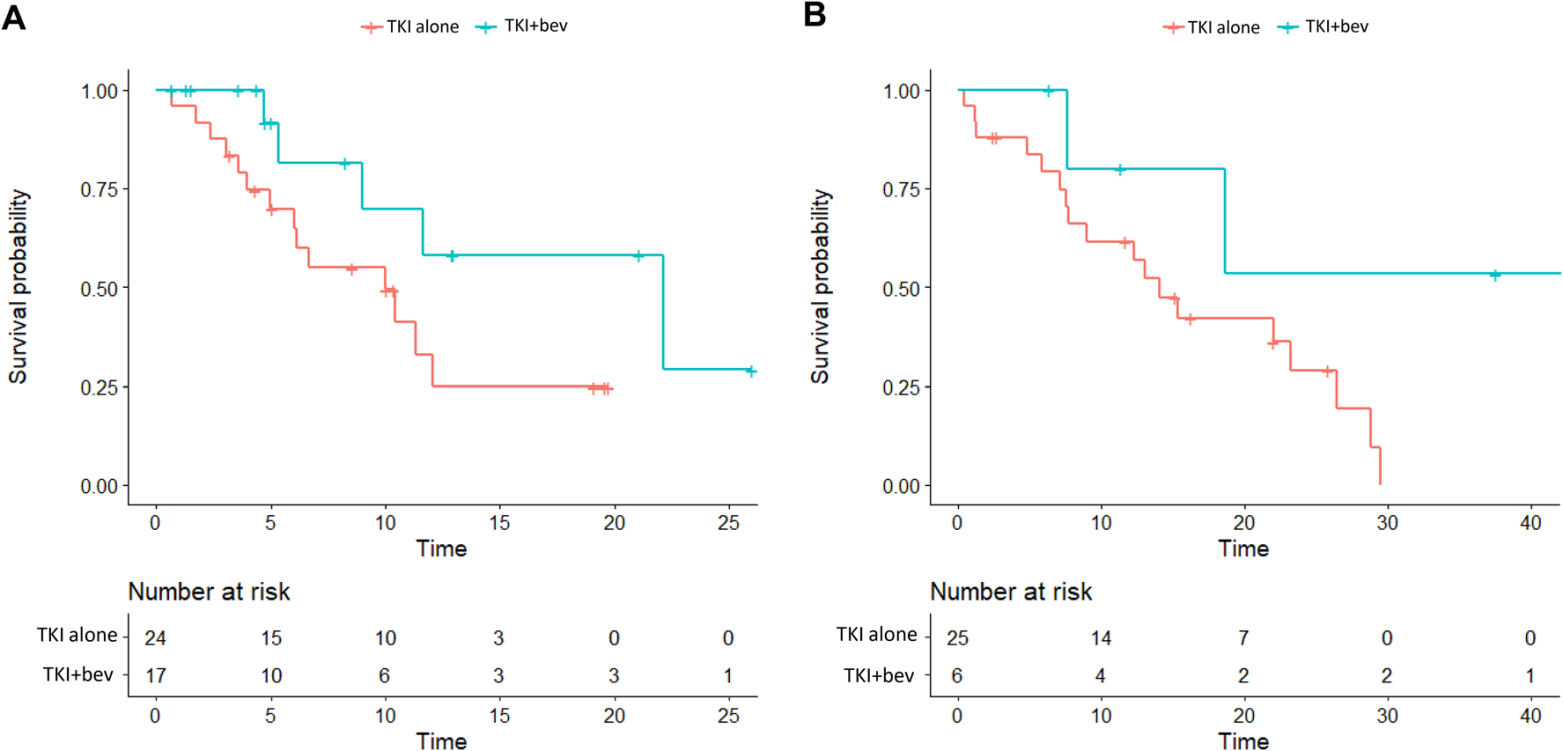
OS of post-progression (**A**) osimertinib treatment and (**B**) chemotherapy between EGFR-TKI plus bevacizumab and EGFR-TKI monotherapy groups. *bev* bevacizumab, *TKI* tyrosin kinase inhibitor.

In the original cohort of 310 patients, 119 (38.4%) patients underwent a tissue and/or liquid biopsy for the diagnosis of *EGFR* T790M mutation where 87 (73.2%) patients were T790M-positive. The T790M-positive rate did not significantly differ between the EGFR-TKI plus bevacizumab and EGFR-TKI monotherapy groups (66.7% vs. 75.0%, p = 0.460). Forty-one (31.1%) and 31 (23.5%) patients of the propensity-score matched cohort, upon disease progression, received subsequent osimertinib treatment or chemotherapy, respectively. Patients of EGFR-TKI plus bevacizumab and EGFR-TKI monotherpay groups demonstrated post-progression osimertinib OS of 22.1 and 10.0 months and post-progression osimertinib 1-year OS rates of 58.2% (95% CI 33.6–100.0%) and 33.0% (95% CI 16.1–67.3%; Fig. 4A), respectively. The post-progression chemotherapy OS were 44.9 and 14.1 months and post-progression chemotherapy 1-year OS rates were 80.0% (95% CI 51.6–100.0%) and 61.6% (95% CI 44.7–84.9%; Fig. 4B) in EGFR-TKI plus bevacizumab and EGFR-TKI monotherpay groups, respectively.

## Discussion

The present study demonstrated a superior OS of first-generation EGFR-TKI plus bevacizumab compared to first-generation EGFR-TKI monotherapy in a real-world cohort of *EGFR*-mutant NSCLC patients with sensitizing mutation. Patients with brain metastasis received additional OS benefit from EGFR-TKI plus bevacizumab. The development of secondary T790M was similar between the two groups. The OS of post-progression treatment, in terms of osimertinib or chemotherapy, in EGFR-TKI plus bevacizumab group were significantly improved compared to single-agent EGFR-TKI group.

Although the present study failed to observe PFS benefit of EGFR-TKI plus bevacizumab in *EGFR*-mutant NSCLC, the OS outcome was significantly improved in patients receiving the regimen. This result was mainly associated with improved efficacies of post-progression treatments after the exposure of EGFR-TKI plus bevacizumab in patients with brain metastasis. Previously, a number of studies have demonstrated satisfactory efficacies of bevacizumab in selected patients with heavily pre-treated symptomatic brain metastasis from advanced solid tumors^24–26^. Studies which primarily focused on NSCLC-related brain metastasis also revealed bevacizumab as a viable option that improved intracranial response, stabilized neurological symptoms and reduced systemic corticosteroid use^27–29^. Recently, a retrospective study analysed a cohort of *EGFR*-mutant NSCLC patients with multiple brain metastasis who received either EGFR-TKI plus bevacizumab or single-agent EGFR-TKI where significant PFS and OS benefits were both observed in the EGFR-TKI plus bevacizumab arm^30^. In line with these findings, the present study also demonstrated EGFR-TKI plus bevacizumab provided significant OS benefit in brain metastasis patients who have consisted of approximately 60% of subjects of the propensity-score matched cohort. Overall, patients with brain metastasis represented the major source of OS benefit generated by EGFR-TKI plus bevacizumab in present study. In contrary, these patients were excluded in the JO25567 study and consisted of 32% of the overall population in the NEJ 026 study which may be partly associated with the negative OS results in these studies.

Interestingly, the benefit of bevacizumab treatment in patients with brain metastasis did not seem to be CNS-restricted. Tao et al. specifically investigated a cohort of *EGFR*-mutant NSCLC patients with multiple brain metastasis and thereby demonstrated that EGFR-TKI plus bevacizumab, compared to EGFR-TKI monotherapy, showed improvement of both intracranial and systemic tumor responses^30^. In present analysis, although the cumulative incidence of CNS and systemic progression over time did not statistically differ between the EGFR-TKI plus bevacizumab and EGFR-TKI monotherapy groups, the early emergence of CNS and systemic progressions were both suppressed in EGFR-TKI plus bevacizumab group and thereby the both types of progression were delayed to occur beyond 8 to 9 months of the treatment. This finding, in association with previous study, likely suggested an improved extracranial tumor control in bevacizumab-treated patients who had intracranial metastasis. Altogether, the bevacizumab treatment-associated CNS and CNS-sparing benefits in patients of brain metastasis may jointly foster a condition that favors the efficacy of subsequent treatments. This was demonstrated in the present analysis that the OS of second-line osimertinib and chemotherapy were both improved in EGFR-TKI plus bevacizumab group compared to single-agent EGFR-TKI.

In addition, other factors related to post-progression treatment may also have impact on OS outcome. Because a cross-over design was allowed in the NEJ 026 study, 28.6% patients of the single-agent erlotnib arm have received subsequent bevacizumab treatment upon disease progression whereas, in present study, only 3.4% of patients who received monotherapy EGFR-TKI have undergone post-progression bevacizumab treatment. Of note, in this analysis, 18.6% of patients who received EGFR-TKI plus bevacizumab continued bevacizumab treatment beyond progression whereas the rate was only 4.5% of the erlotinib plus bevacizumab arm in the NEJ 026 study. The differential rate of bevacizumab treatment beyond progression may also have some impact on OS as previous AvaALL trial has demonstrated a minor benefit of bevacizumab treatment beyond progression compared to standard-of-care alone^31^.

As a new recommended first-line treatment for advanced *EGFR*-mutant NSCLC, third-generation EGFR-TKI osimertinib provided an improved OS compared to gefitinib/erlotinib. In the *post-hoc* analysis, cohort of *EGFR* exon 19 deletion patients was the major source that contributed to the OS benefit whereas cohort of *EGFR* L858R patients generally received minimal treatment benefit from osimertinib. In contrary, the *post-hoc* analysis of NEJ 026 study revealed that bevacizumab likely offered additional benefit to the cohort of *EGFR* L858R patients and this finding has also been reported in real-world patients^32^. The present study also observed a trend of OS benefit in *EGFR* L858R patients who received combination of EGFR-TKI and bevacizumab. Hence, these findings may warrant further investigation of bevacizumab treatment in patients of *EGFR* L858R genotype.

Previously, pre-clinical study demonstrated activity of bevacizumab to *EGFR* T790M mutant and a randomized phase II trial also showcased an improved PFS in patients with de novo T790M mutation treated by erlotinib plus bevacizumab^33,34^. However, the potential activity of anti-angiogenesis agent to *EGFR* T790M mutant observed previously did not seem to suppress the development of secondary T790M mutation when it was administered simultaneously with EGFR-TKI. This has been observed in the NEJ 026 and RELAY studies in which the rates of secondary T790M were similar between the anti-angiogenesis agent plus erlotinib and monotherapy erlotinib groups^35,36^. Similarly, only a numerically lower secondary T790M rate by 8.3% was observed in EGFR-TKI plus bevacizumab group in the present study. Nevertheless, more real-world data is still required to clarify the impact regarding treatment of anti-angiogenesis agent and the development of secondary T790M. The limitation of the present study, firstly, is the potential bias due to retrospective nature per se. Therefore, the treatment-related complications of bevacizumab such as hypertension, peripheral edema and bleeding which may be associated with poor prognosis could not be recorded as comprehensive as a prospective study. Secondly, a small portion (9.8%) of patients of the propensity-score matched cohort received gefitinib, instead of erlotinib, as the first-generation EGFR-TKI and thus increased the data heterogeneity. However, an earlier large scale meta-analysis has suggested similar efficacy of the two drugs^37^ and thus the heterogeneity is likely acceptable.

In conclusion, this study showcased an improved OS of EGFR-TKI plus bevacizumab in a real-world cohort of patients with advanced *EGFR*-mutant NSCLC in which patients with brain metastasis received most benefit from the regimen. Further study of bevacizumab treatment in brain metastasis-specific cohort of advanced NSCLC patients is warranted.
